# Genetic Modification of the Soybean to Enhance the β-Carotene Content through Seed-Specific Expression

**DOI:** 10.1371/journal.pone.0048287

**Published:** 2012-10-31

**Authors:** Mi-Jin Kim, Jae Kwang Kim, Hye Jeong Kim, Jung Hun Pak, Jai-Heon Lee, Doh-Hoon Kim, Hong Kyu Choi, Ho Won Jung, Jeong-Dong Lee, Young-Soo Chung, Sun-Hwa Ha

**Affiliations:** 1 Department of Genetic Engineering, Dong-A University, Busan, Republic of Korea; 2 National Academy of Agricultural Science, Rural Development Administration, Suwon, Republic of Korea; 3 School of Applied Biosciences, Kyungpook National University, Daegu, Republic of Korea; Cairo University, Egypt

## Abstract

The carotenoid biosynthetic pathway was genetically manipulated using the recombinant *PAC* (*Phytoene synthase-2A-Carotene desaturase*) gene in Korean soybean (*Glycine max* L. cv. Kwangan). The *PAC* gene was linked to either the β-conglycinin (β) or CaMV-35S (35S) promoter to generate *β-PAC* and *35S-PAC* constructs, respectively. A total of 37 transgenic lines (19 for *β-PAC* and 18 for *35S-PAC*) were obtained through *Agrobacterium*-mediated transformation using the modified half-seed method. The multi-copy insertion of the transgene was determined by genomic Southern blot analysis. Four lines for *β-PAC* were selected by visual inspection to confirm an orange endosperm, which was not found in the seeds of the *35S-PAC* lines. The strong expression of *PAC* gene was detected in the seeds of the *β-PAC* lines and in the leaves of the *35S-PAC* lines by RT-PCR and qRT-PCR analyses, suggesting that these two different promoters function distinctively. HPLC analysis of the seeds and leaves of the T_2_ generation plants revealed that the best line among the *β-PAC* transgenic seeds accumulated 146 µg/g of total carotenoids (approximately 62-fold higher than non-transgenic seeds), of which 112 µg/g (77%) was β-carotene. In contrast, the level and composition of the leaf carotenoids showed little difference between transgenic and non-transgenic soybean plants. We have therefore demonstrated the production of a high β-carotene soybean through the seed-specific overexpression of two carotenoid biosynthetic genes, *Capsicum* phytoene synthase and *Pantoea* carotene desaturase. This nutritional enhancement of soybean seeds through the elevation of the provitamin A content to produce biofortified food may have practical health benefits in the future in both humans and livestock.

## Introduction

Plant carotenoids, a subfamily of isoprenoids, are a group of natural pigments that are synthesized in plastids and have biologically indispensable roles i.e. the chloroplast carotenoids protect the photosynthetic apparatus from excess light energy and the chromoplast carotenoids of fruits, seeds and flowers act as pollinators and vehicles for seed dispersion [1], [2]. Carotenoids are also precursors of abscisic acid (ABA), which regulates plant growth, seed dormancy, embryo development and stress responses [3]. In addition, some carotenoids, including β-carotene, are precursors of vitamin A which is essential for human health. Vitamin A deficiency (VAD) leads to severe clinical symptoms associated with night blindness, xerophthalmia and breakdown of the human immune system [4]. Fatal instances of VAD in infants and pregnant women have been a major problem in developing countries where there is a dependence on a single staple crop for sustenance [5]. To overcome VAD through food intake, plant transgenics has been used to increase the levels of β-carotene (provitamin A) in canola, tomato, rice, potato, carrot and maize [6]–[12].

Among the best known examples of a transgenic crop that was engineered to produce high levels of β-carotene is ‘Golden Rice’ which was generated using two cassettes for the expression of the phytoene synthase (*Psy*) and carotene desaturase (*CrtI*) genes [8], [9]. Recently, ‘New Golden Rice’ has been reported in which two bicistronic recombinant genes *Psy-2A-CrtI* (*PAC*) and *Psy-IRES-CrtI* (*PIC*), involving either 2A or IRES sequences to simultaneously express *Psy* and *CrtI*, were compared and the 2A sequence (*PAC*) was shown to be more potent [13]. The *PAC* gene has driven the expression of the two genes required for β-carotene production through a single transcript and is therefore potentially applicable to the development of other β-carotene transgenic crops including the soybean.

Soybean [*Glycine max* (L.) Merrill] is one of the world’s most important crops as not only an important source of oil and protein, but also secondary metabolites. Improvements to the functional aspects of the soybean have been previously investigated using transformation techniques [14], [15]. Since transgenic soybean plants have been steadily developed since the 1980s, the introduction of insect and herbicide resistance traits via transformation with a synthetic Bt cryIAc or 5-enolpyruvylshikimate-3-phosphate synthase gene, respectively, has greatly enhanced soybean production worldwide. Moreover, improvements in the efficiency of soybean transformation techniques have been continuously required for functional genomics research and crop improvement applications. Thus far, the most efficient method of soybean transformation has been found to be the *Agrobacterium*-mediated system based on the cotyledonary-node (CN) [16]–[18]. An improved CN method using half-seed explants has now been reported to reduce the time required for seed germination and precise shoot cutting [19].

In our present study, we genetically manipulated the carotenoid biosynthetic pathway in soybean plants using an efficient *Agrobacterium*-mediated transformation method and the *PAC* recombinant gene. We were thereby able to produce high β-carotene contents in the transformants which exceeded the levels previously reported in transgenic rice endosperm when the same gene had been used. We here describe the effectiveness of using the *PAC* gene in soybean to produce high levels of β-carotene when expressed in a seed-specific manner.

## Results

### Korean Soybean Transformants using an Efficient *Agrobacterium*-mediated Method

**Figure 1:**
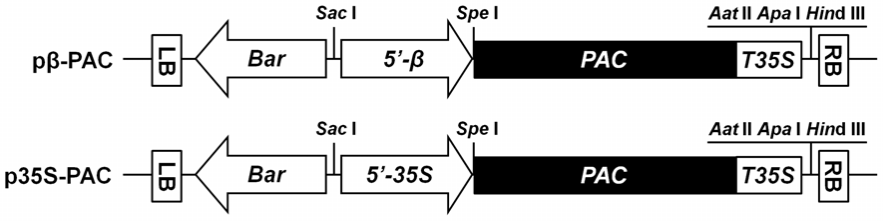
Schematic representation of the binary vectors used in the soybean transformations. Two vectors contained the same recombinant *PAC* gene as a closed box downstream of a soybean seed specific β-conglycinin promoter and a cauliflower mosaic virus 35S promoter, respectively. The remaining construct components had the same configuration, consisting of the *Bar* cassette to express the DL-phosphinothricin resistance gene driven by the 35S promoter and the cauliflower mosaic virus 35S terminator. LB and RB, left and right borders for *Agrobacterium*-mediated transformation, respectively; *5′-β*, a soybean seed specific β-conglycinin promoter; *5′-35S*, a cauliflower mosaic virus 35S promoter; *PAC*, a recombinant *Psy-2A-Tp-CrtI* gene; T35S, a cauliflower mosaic virus 35S terminator.

**Figure 2:**
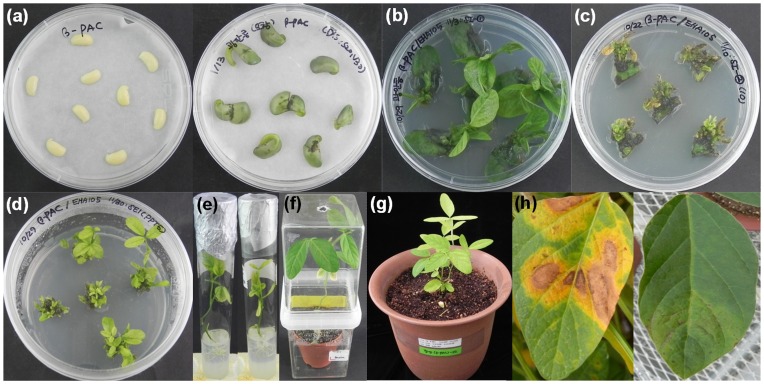
*Agrobacterium*-mediated transformation of half-seed explants and regeneration. (a) Half seed explants immediately after infection (left) and at five days after inoculation (right). (b) Shoot induction medium without DL-phosphinothricin. (c) Shoot induction medium containing DL-phosphinothricin (10 mg/L) for Basta® selection. (d) Shoot elongation medium with DL-phosphinothricin (5 mg/L). (e) Rooting medium. (f) Acclimated putative transgenic plant in a small pot. (g) Transgenic plant growing in a large pot. (h) Leaf painting assay showing wild type plant sensitivity (left) and transgenic plant resistance (right) to Basta® at five days after treatment with DL-phosphinothricin (100 mg/L).

To enhance the nutritional characteristics of soybean plants through increased levels of β-carotene, the carotenoid biosynthetic pathway was genetically engineered to express the recombinant *PAC* gene using two different promoters: β-conglycinin for seed-specific expression and CaMV-35S for constitutive over-expression. The corresponding *β-PAC* and *35S-PAC* plasmids are depicted schematically in Figure 1. In the plant transformations, half-seeds of the Korean soybean cultivar (*Glycine max* L. cv. Kwangan) were co-cultivated with *Agrobacterium* strains harboring either *β-PAC* or *35S-PAC* (Fig. 2a). Prior to this co-cultivation for five days, the inoculation of *Agrobacterium* into half seeds was performed using three additional steps, dipping with a high concentration of *Agrobacterium* solution, sonication and the use of a vacuum. Explants were placed onto the shoot induction media (SIM) without the herbicide PPT (Fig. 2b). Normal shoot formation and proliferation were inducted in SIM-containing PPT for two weeks (Fig. 2c). Another two weeks later, trimmed shoots that had a removed callus were transferred to shoot elongation media (SEM) with PPT (Fig. 2d). The elongated shoots after four to six weeks were excised and transferred to root induction medium (RIM) (Fig. 2e). At this time, the leaf size of shoots is quite critical as relatively larger leaves result in better root formation. Rooted plantlets were transplanted to small pots containing a mixture of soil and vermiculite and then placed into a closed magenta jar for acclimation (Fig. 2f). The resultant T_0_ plantlets were then transplanted in soil to large pots and grown in a greenhouse (Fig. 2g).

Herbicide-resistance in transgenic soybean plants was examined using a leaf painting assay in which the surfaces of two trifoliate leaves were brushed with PPT solution (100 mg/L). At one week after this herbicide application, healthy transgenic leaves were discernible from the non-transgenic leaves as they did not wilt and die (Fig. 2 h). The whole procedure for obtaining transgenic soybean plants i.e. from half-seed preparation to the harvesting of T_1_ seeds, took seven months. Four soybean transformation trials were attempted for this study and 200 seeds were used in each experimental trial with the final efficiency of soybean transformation measured at about 5% (data not shown). A total of 43 putative transgenic soybean plants (24 for *β-PAC* and 19 for *35S-PAC*) were produced and cultivated to produce T_1_ seeds in the greenhouse.

### Inspection of Seed Color Change through the Introduction of the Recombinant *PAC* Gene

**Figure 3:**
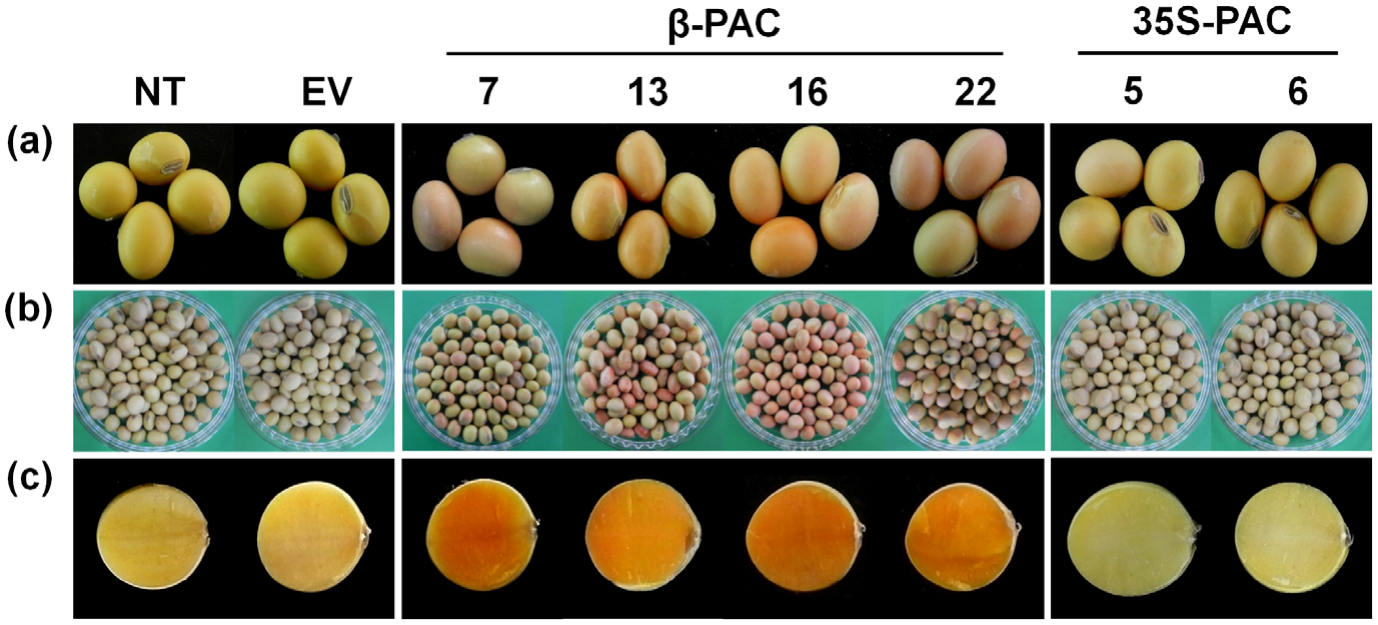
Photographs of *β-PAC* and *35S-PAC* transgenic soybean seeds. (a) T_1_ seeds. (b) T_2_ seeds. (c) Cross sections of T_2_ seeds. NT, non-transgenic plants; EV, empty vector-transgenic plants.

Among the T_1_ seeds obtained, the seed color change in four *β-PAC* lines from yellow to orange was determined by visual inspection (Fig. 3a). These seeds were preferentially selected as *β-PAC* transgenic lines whilst none of *35S-PAC* lines showed any seed color changes. The maintenance of the orange seed color was determined for the T_2_ seed generation (Fig. 3b) and this color intensity was found to be stronger in the T_2_ seeds from an analysis of their cross sections, suggesting a higher accumulation of carotenoids by gene homozygosis as the progress of generation (Fig. 3c). In contrast, none of the T_2_ seeds in any of the *35S-PAC* transgenic lines showed any changes in seed color, as found also in the non-transgenic and empty-vector transgenic soybean seeds.

### Integration and Expression of Transgenes in Soybean Plants

**Figure 4:**
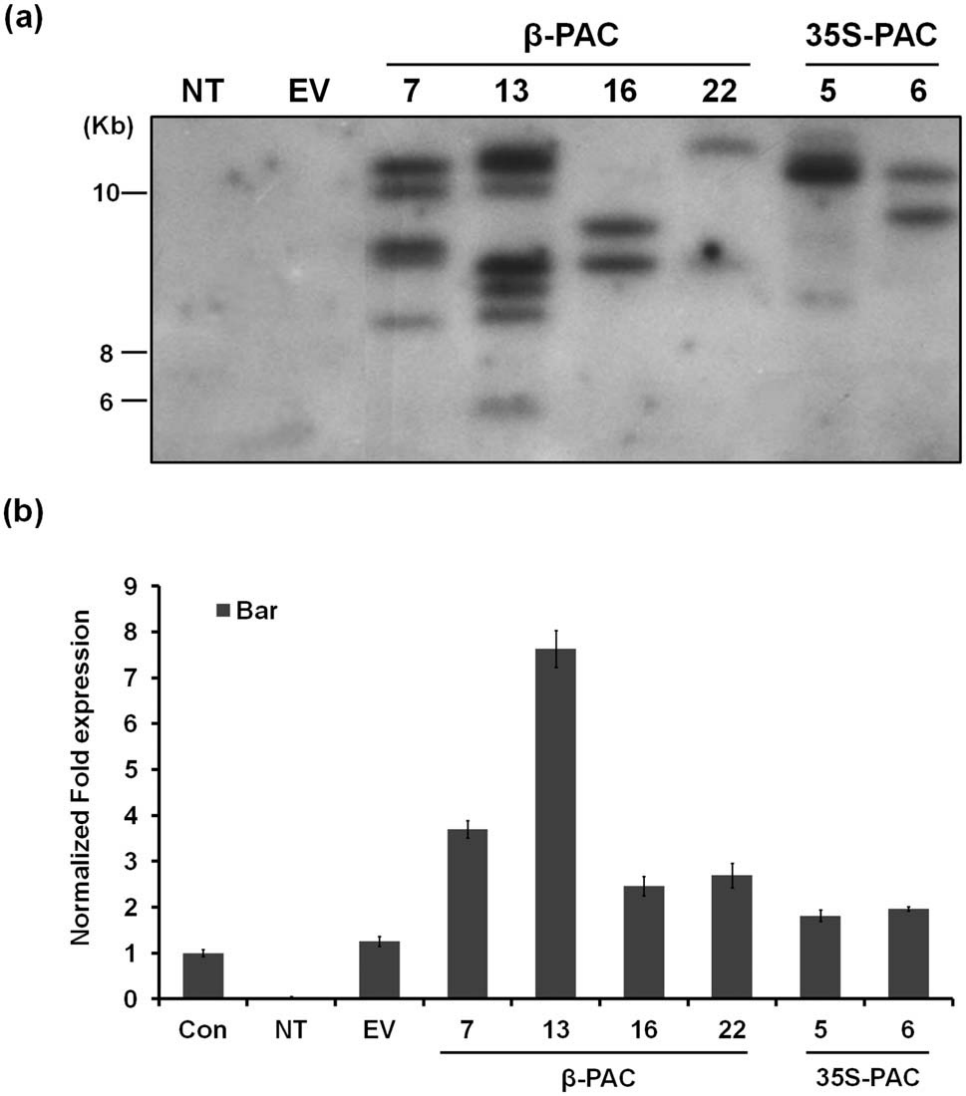
Determination of insertion events of *β-PAC* and *35S-PAC* transgenes. (a) Genomic Southern blot analysis was performed with genomic DNAs from each leaf tissues and a *PAC* probe. The DNA molecular size markers are indicated on the left. (b) Quantitative real-time PCR analysis was carried out with the same genomic DNAs and a *Bar* primer set. Con, transgenic plant that its single *Bar* gene insertion was already confirmed NT, non-transgenic plants; EV, empty vector-transgenic plants.

To verify transgene integration in our transformed soybean plants, leaf genomic DNA extracts were prepared from all putative T_0_ transformants and subjected to PCR analysis using primers designed to amplify the coding sequences of the *PAC* and *Bar* genes, respectively. The *β-PAC* and *35S-PAC* transformants were further verified by amplifying the DNA region between the β-conglycinin promoter and *PAC* gene or 35S promoter and *PAC* gene, respectively. The intactness of the introduced gene regions was examined by further genomic PCR analysis (Figure S1 and S2) and successful introduction of a complete transgene was confirmed in almost all of our putative transformants. Five out of 24 *β-PAC* and 1/19 of the *35S-PAC* lines had missing regions of the integrated gene. A final total of 37 transgenic lines (19 *β-PAC* and 18 *35S-PAC*) were thus obtained using *Agrobacterium*-mediated transformation. To determine the number of transgene insertion events in these lines, genomic Southern blotting of four *β-PAC* and two *35S-PAC* lines was performed using a *PAC* probe (Fig. 4a). We confirmed that each of these six transgenic lines contained multiple events of their respective transgene. To further estimate the number of T-DNA integration, quantitative real-time PCR (qRT-PCR) with the same genomic DNA and a set of *Bar* primer was carried out (Fig. 4b). Except the *35S-PAC* 5 line that showed the different results, ambiguous in Southern blotting with a *PAC* gene and two in qRT-PCR with a *Bar* gene, rest of all showed the same patterns of transgene presence (two in *β-PAC* 16, *β-PAC* 22 and *35S-PAC* 6 and over four in *β-PAC* 7 and *β-PAC* 13).

**Figure 5:**
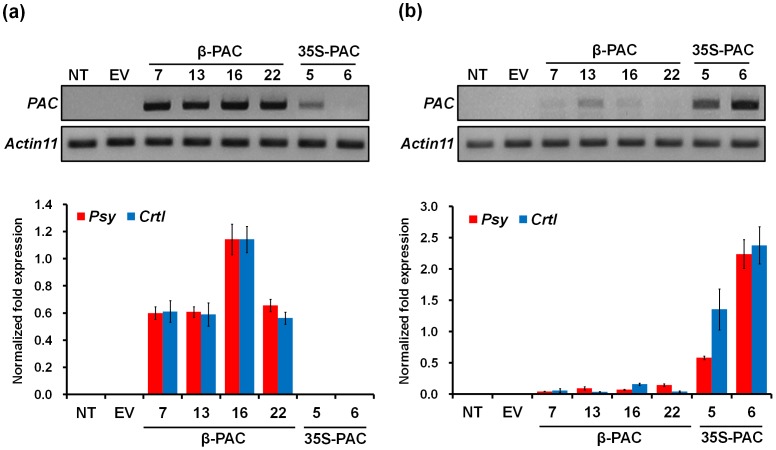
Reverse-transcriptase PCR (upper) and quantitative real-time (bottom) analyses of *β-PAC* and *35S-PAC* transgenic soybean lines. (a) Seeds. (b) Leaves. NT, non-transgenic plants; EV, empty vector transgenic plants. The 7, 13, 16 and 22 *β-PAC* transgenic lines and 5 and 6 *35S-PAC* transgenic lines were analyzed at the T_2_ generation. The *Actin11* gene was used as the normalization control for both the seed and leaf RNA levels.

To analyze the expression level of the *PAC* transgene for the different promoters, reverse transcriptase-PCR and qRT-PCR analyses were performed using RNAs extracted from seed and leaf tissues of the *β-PAC* and *35S-PAC* transgenic soybean lines (Fig. 5). In the seeds, both PCR assays revealed a much stronger expression of the *PAC* gene in the *β-PAC* compared with the *35S-PAC* transgenic lines, indicating that authentic seed-specific expression is driven by the β-conglycinin promoter (Fig. 5a). On the other hand, the levels of *PAC* gene expression in leaves were found to be higher in the *35S-PAC* lines (Fig. 5b). We therefore obtained two kinds of transgenic soybean that expressed the same *PAC* gene in either seeds or leaves. The effects of this organ-specific expression on the total carotenoid levels and flux changes in the carotenoid metabolic pathway were then further examined.

### Carotenoid Composition in the *β-PAC* and *35S-PAC* Transgenic Soybean Plants

**Figure 6:**
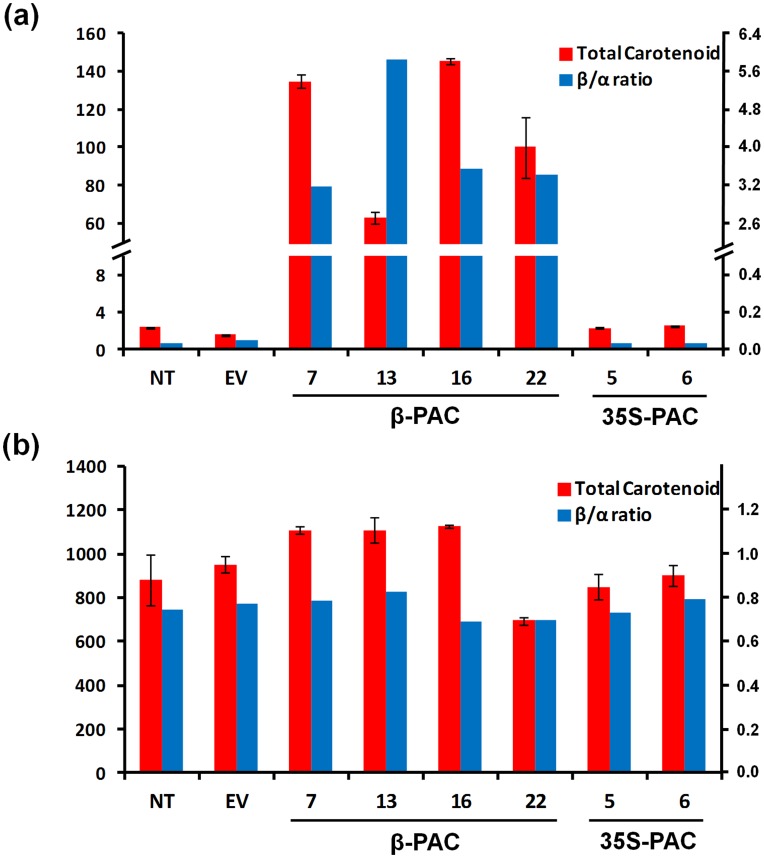
Total carotenoid content and β/α ratio analysis of *β-PAC* and *35S-PAC* transgenic soybean plants. (a) Seeds. (b) Leaves. Total carotenoid levels were calculated as the sum of eight carotenoid subtype levels i.e. violaxanthin, antheraxanthin, lutein, zeaxanthin, α-cryptoxanthin, β-cryptoxanthin, α-carotene and β-carotene. The β-carotenoids include violaxanthin, antheraxanthin, zeaxanthin, β-cryptoxanthin and β-carotene and α-carotenoids include lutein and α-carotene in the determination of the β- to α- carotenoid ratios. Values (µg/g dry weight) are the means of three replicates. Error bars represent the standard deviations.

Using HPLC analysis, we measured the total content and composition of the carotenoids in the seeds and leaves of T_2_ generation *β-PAC* and *35S-PAC* transgenic soybean plants (Table S1). In the seeds, the *β-PAC* soybean lines produced very high levels of total carotenoids (an average of 110.7 g/g), which were 45-fold higher than these levels in the *35S-PAC* lines (an average of 2.5 g/g) and the negative control non-transgenic (2.4 g/g) and empty vector-transgenic (1.6 g/g) soybean plants. The average ratio of β- to α-carotenoids in the *β-PAC* lines (3.6) was found to be markedly higher than either the *35S-PAC* lines (0.03) or non-transgenic lines (0.03). Hence, the use of the seed-specific β-conglycinin promoter successfully yielded a high accumulation of β-carotenoids in soybean seeds (Fig. 6a). This result suggested that the preference for the α-branch in the soybean seed was entirely changed to the β-branch through the *PAC* gene introduction.

The total carotenoid levels in the leaves was slightly higher in the *β-PAC* (an average of 1,008 g/g) compared with the *35S-PAC* (an average of 875 g/g) transgenic plants and negative controls (882 and 951 g/g, respectively). However, the average ratios of β- to α-carotenoids among the negative controls (0.74 and 0.77) and transgenic plants (*35S-PAC*, 0.76; *β-PAC*, 0.75) showed simillarities. These data suggested that exogenous *PAC* gene expression in the soybean leaves does not affect the total carotenoid level or the β- to α-carotenoid ratio regardless of the specificity of the promoter used (Fig. 6b).

**Figure 7:**
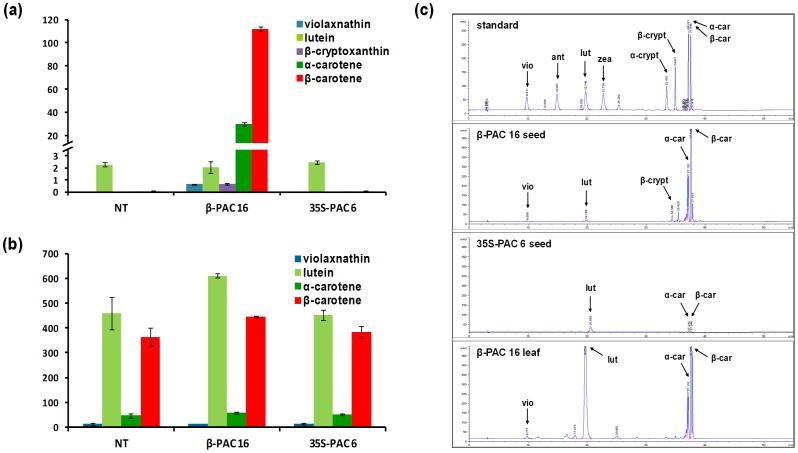
Carotenoid composition of *β-PAC* and *35S-PAC* transgenic soybean plants and their HPLC chromatograms. (a) Seeds. (b) Leaves. The composition of individual carotenoids is shown for representative lines for *β-PAC* (line 16) and *35S-PAC* (line 6). More detailed results of carotenoid composition analyses are listed in Table S1. (c) Representative HPLC chromatograms of transgenic seeds from *β-PAC* and *35S-PAC* lines and transgenic leaves of *β-PAC* lines. Carotenoid standards included violaxanthin (vio), antheraxanthin (ant), lutein (lut), zeaxanthin (zea), α-cryptoxanthin (α-crypt), β-cryptoxanthin (β-crypt), α-carotene (α-car) and β-carotene (β-car). Values (µg/g dry weight) are the mean of three replicates. Error bars represent the standard deviations.

Among individual components of the carotenoids in non-transgenic soybean seeds (Table S1), lutein (96.6% of total carotenoid) was found to be far more abundant than the other carotenoids, including β-carotene (2.5%) and α-carotene (0.8%). There was no lycopene, β-cryptoxanthin, zeaxanthin, violaxanthin or antheraxanthin detected in the seed endosperm of these controls. The seeds of *35S-PAC* transgenic soybean plants showed the same compositions and levels of carotenoids as both negative control plants, further suggesting that *PAC* gene expression via the 35S promoter has no impact on carotenoid accumulation in seeds. In contrast, the seed endosperms in the *β-PAC* transgenic soybean plants showed large increases in β-carotene (28-fold) and α-carotene (25-fold) compared with the *35S-PAC* endosperms. Unlike the other plants tested, β-carotene was the predominant component (77% of the total), followed by α-carotene (20%), in the *β-PAC* transgenic seeds. The level of lutein remained unchanged in the *β-PAC* seeds but the two β-carotenoid components, β-cryptoxanthin and violaxanthin, were found to be newly accumulated (Fig. 7a).

In the leaves of the non-transgenic soybean plants, the main component of soybean carotenoids was found to be lutein (52% of total carotenoid), followed by β-carotene (41%), α-carotene (5%) and violaxanthin (1.4%). These same ratios were found in the leaves of all transgenic soybean plants including *35S-PAC* and *β-PAC* even through the levels of lutein and β-carotene slightly increased in the *β-PAC* leaves (Fig. 7b).

### Tocopherol and Phytosterol Compositions in the *β-PAC* and *35S-PAC* Transgenic Soybean Seeds

**Figure 8:**
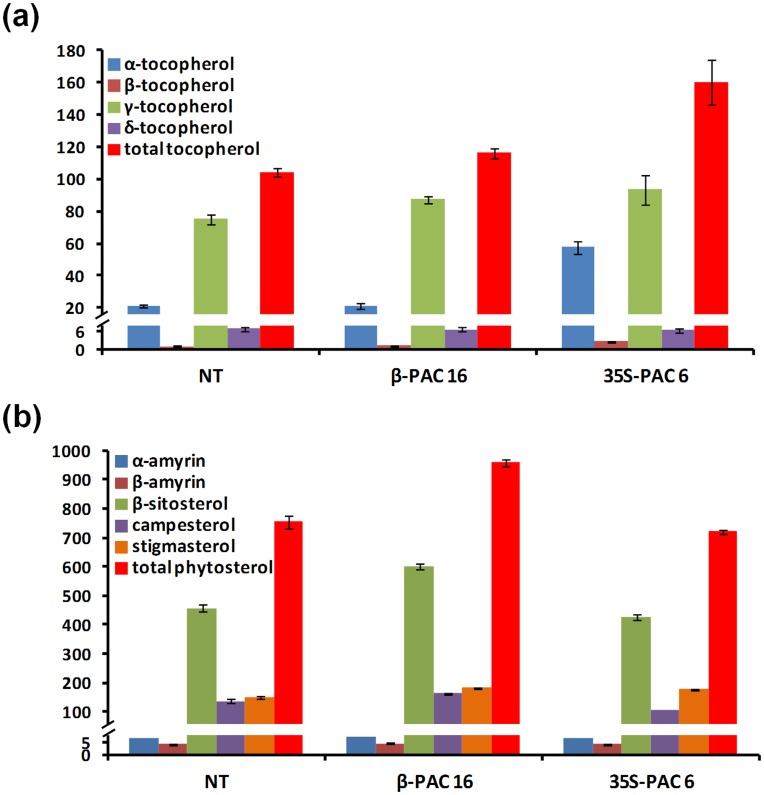
Tocopherol and phytosterol composition in the seeds of *β-PAC* and *35S-PAC* transgenic soybean plants. (a) Tocopherols. (b) phytosterols. The individual compositions in representative lines for *β-PAC* (line 16) and *35S-PAC* (line 6) are shown. Values (µg/g dry weight) are the mean of three replicates. Error bars represent the standard deviations.

To examine the effects of the exogenous expression of *PAC* on some of the lipophilic metabolic processes associated with the carotenoid pathway in soybean seeds, we measured the levels of tocopherols and phytosterols using gas chromatography-time-of-flight mass spectrometry (GC-TOFMS) analysis. The mean total tocopherol levels, including α-, β-, γ- and δ-tocopherols, were similar in the *β-PAC* seeds but were 1.5-fold higher in the *35S-PAC* seed, compared with the non-transgenic control (Fig. 8a). Of particular note, the α-tocopherol level was about 3-fold higher in *35S-PAC* line than in the *β-PAC* line and the non-transgenic control. In addition, quantification of phytosterols including α-amyrin, β-amyrin, β-sitosterol, campesterol and stigmasterol showed 1.3 times higher of total level due to the increase of β-sitosterol in *β-PAC* than *35S-PAC* and non-transgenic control (Fig. 8b). These results indicate that the expression of carotenoid biosynthetic genes in soybean plants differently regulates the levels of two lipophilic products synthesized via pathways linked to the isoprenoids, including the carotenoids. To have more confidence, the number of transgenic lines to be analyzed two lipophilic compounds was increased (Figure S3). As results, two of all *35S-PAC* lines showed the increase of total tocopherols (particularly in α-tocopherol) and four of all *β-PAC* lines showed the increase of total phytosterols (particularly in β*-*sitosterol) in seeds. It suggested that the consistence pattern of two lipophilic components levels among transgenic lines might be attributed to the expression of *PAC* transgene in soybean plants.

### Radical Scavenging Activity of the *β-PAC* and *35S-PAC* Transgenic Soybean Seeds

To compare the antioxidant activity of soybean seeds according to carotenoid accumulation, radical scavenging effect using 2,2′-diphenyl-1-picrylhydrazil (DPPH), a stable free radical, was examined. Based on the principle that the maximum absorbance at 517 nm fades rapidly when DPPH encounters proton-radical scavengers, the antioxidant effect is proportional to the disappearance of DPPH, by presenting the result as EC_50_ that is the amount of an antioxidant that causes a decrease in an initial DPPH concentration by 50% [20]. Through this DPPH assay, the radical scavenging effect using the methanol extracts of *β-PAC* and *35S-PAC* transgenic soybean seeds were measured (Table 1). As results, the radical scavenging activity of *35S-PAC* transgenic soybean seeds showed the similar level to two control plants of NT and EV. While, All *β-PAC* transgenic soybean seeds showed higher radical scavenging activity than others. It strongly suggested that the accumulation of carotenoids including β-carotene might be responsible for the antioxidant activity through radical scavenging.

**Table 1:** DPPH radical scavenging activity of *β-PAC* and *35S-PAC* transgenic soybean seeds.

Sample	EC_50_ value ± SD (mg/10 mL)
α-Tocopherol	0.12±0.01
NT	68.64±2.87ab
EV	75.25±3.97a
β-PAC 7	59.41±2.91cd
β-PAC 13	40.21±2.92f
β-PAC 16	42.97±0.75ef
β-PAC 22	52.02±2.34de
35S-PAC 5	72.75±1.63ab
35S-PAC 6	65.19±3.03bc

The values of the DPPH are the mean of three determinations ± standard deviation (SD). Different letters represent significant (*P*<0.05) differences between means according to ANOVA combined with Duncan’s multiple-range test.

## Discussion

Value improvements to the commercially important soybean plant using biotechnology have increased with the development of better transformation technologies. Since the CN method was applied to 28 diverse cultivars and/or genotypes of the soybean [17], improvements to this protocol have been suggested using half-seed explants of the Williams82 soybean cultivars (4.5%) which have been reported to increase the efficiency of transformation [19]. In our current study, we employed this adjusted CN method using half-seeds of the Korean soybean cultivar Kwangan in a three step treatment procedure (high concentration of *Agrobacterium* solution, sonication and vacuum) during the early stages of *Agrobacterium* inoculation. This approach has been validated in a previous study using 90 Korean soybean genotypes [21]. However, further optimization of these transformation conditions are still required to reduce the integration event numbers of transgene in the soybean genome, which we detected in our present experiments (Fig. 4). High numbers of the target gene integration may cause undesirable phenotypic outcomes due to gene silencing or an uncontrollable segregation of the transgenes. For example, the *β-PAC* line 13 in our current series was found to contain seven transgenes (Fig. 4) but showed the lowest carotenoid levels among the panel of *β-PAC* lines tested (Fig. 6a and Table S1). This was in spite of the comparable levels of transgene expression between this and the other lines (Fig. 5a).

We also investigated the agronomical traits of our transgenic soybean plants under field conditions using T_2_ heterozygous lines. Several yield-related characteristics such as flowering time, the number of branch and nodes, and the 100 seed weight showed no differences between the transgenic and non-transgenic plants. In addition, phenotypes such as flower color, pubescence, leaf shape and plant height, further indicated that the *PAC* transgene has no significant influence on morphological traits in the soybean (data not shown).

The *PAC* recombinant gene expressed under the control of the endosperm-specific rice globulin promoter has been previously reported to generate ‘Korean Golden Rice’ with an accumulation of carotenoids [13]. The merits of bicistronic expression in plants have been described previously as an effective alternative to the simultaneous introduction of multiple transgenes [22], [23]. We here report that bicistronic *PAC* gene expression using the seed-specific β-conglycinin promoter achieved much greater success in terms of accumulated β-carotene (112.1 g/g) and total carotenoids (145.5 g/g) in soybean seeds compared with in rice grains (0.64 g/g and 1.35 g/g, respectively). Moreover, this total carotenoid level is four-fold higher than that reported in Golden Rice 2 (36.7 g/g) [9]. A more intense yellow color in the *β-PAC* soybean endosperm (Fig. 3) has an orange hue rather than gold due to the de novo biosynthesis of high amounts of β-carotenoids (β-carotene, β-cryptoxanthin and violaxanthin) and α-carotene (Fig. 7a and Table S1). This exceptional carotenoid metabolic engineering result in a plant storage organ is likely due to the seed-specific expression of the *PAC* transgene using an appropriate promoter (Fig. 5a). In contrast, the constitutive expression of the same *PAC* gene using 35S promoter had no impact on carotenoid accumulation despite strong transgene expression in the leaves (Fig. 5b and 6b). This finding suggested that the carotenoid levels in photosynthetic organs tend to be in equilibrium under forced gene expression conditions, being consistent in the leaf of the citrus lycopene β-cyclase transgenic tomato [24]. The fact that the expressing carotenoid genes using constitutive promoter like 35S has been hard to increase the level of carotenoid in leaf has been also presented in previous study [25].

Further quantification of two lipophilic products synthesized by pathways linked to the carotenoids within the isoprenoid metabolic framework revealed that carotenoid augmentation is accompanied by increases in the phytosterol but not the tocopherol levels (Fig. 8). This is in agreement with a previous proposal that a fully functioning pathway producing plastidic isoprenoids (carotenoids in this study) may enhance the levels of cytoplasmic isoprenoid-like sterols [26]. However, the result that the level of total tocopherols was increased in *35S-PAC* seeds is not easy to view the direct effect of *PAC* gene expression in seeds, considering its very low expression with 35S promoter in soybean seeds (Fig. 5a). When it comes to the strong expression of *PAC* gene with 35S promoter in leaves (Fig. 5b), the tocopherols in *35S-PAC* seeds might be increased under the influence of the over-expression of *PAC* gene in leaves. This excess expression of carotenoid biosynthetic genes in leaves could burden and affect whole plants to be under stress without the carotenoid augmentation in seeds (Fig. 6a) as well as in leaves (Fig. 6b). The study to support that the diverse stresses to plants increased the level of tocopherols has been reported [27]. In the meantime, high antioxidant activity of all *β-PAC* seeds was shown by DPPH assay (Table 1). It was resulted in that the accumulation of β-carotene might be responsible for the antioxidant activity through radical scavenging. It was well accorded with the observance that the variety with higher percentages of β-carotene presented the higher antioxidant activity among six varieties of *Bactris gasipaes* [28].

Together with our previous findings in rice [13], our analysis of the soybean in this study has found that the *PAC* gene can successfully function in both monocotyledons and dicotyledonous plants when expressed under the control of seed-specific promoters. The efficiency in this regard was found to be quite high in our current experiments with the transgenic soybean and showed a 175-fold higher level of β-carotene formation than was found in our previous analyses in transgenic rice plants [13]. Of particular note, we here report one of the highest levels of β-carotene production in soybean seeds (112 g/g DW in *β-PAC* 16 line) when compared with previously reported results in transgenic plants. Some of these results can be summarized as follows: Golden Rice 2 grain (31 g/g DW in SGR2Z1, [9]), maize endosperm (57 g/g DW in Ph-3, [11]) and potato tuber (47 g/g DW in pP-YBI 17, [10]). This current generation of high β-carotene soybean crops has the potential to help alleviate VAD in humans and to provide an important nutrition balance as a biofortified food for livestock with provitamin A and antioxidant activity.

## Materials and Methods

### Vector Construction and *Agrobacterium* Transformation

A recombinant *PAC* gene that was subcloned into the pDONR 221 vector (Invitrogen, Carlsbad, CA), as previously reported [13], and then recombined into the two desired destination vectors, *pB2GW7.0-β-conglycinin* and *pB2GW7.0* (VIB-Ghent University, Ghent, Belgium) using the Gateway cloning method [29]. This produced the constructs *pβ-PAC* and *p35S-PAC*, respectively. In advance of this, the *pB2GW7.0-β-conglycinin* vector had been produced to include the seed-specific *β*-conglycinin promoter [30] instead of the 35S promoter in the parental *pB2GW7.0* vector. The resultant *pβ-PAC* and *p35S-PAC* plasmids were transformed into the *Agrobacterium tumefaciens* strain EHA105 prior to soybean transformation.

### Soybean Transformation

Mature Korean soybean seeds (*Glycine max* L. cv. Kwangan), which was selected through the previous study [21], were utilized in the *Agrobacterium*-mediated transformation experiments. The seeds were surface sterilized for 20 h using chlorine gas produced by mixing 5 ml of 12 N HCl and 100 ml of commercial bleach (12% sodium hypochlorite). One day prior to inoculation, the seeds were soaked in sterile distilled water overnight for about 20 h. For inoculation, previously prepared *Agrobacterium* competent cells were cultured in 200 ml of liquid YEP media containing 25 mg l^−1^ rifampicin, 50 mg l^−1^ spectinomycin for nearly 20 h until an OD_600_ value of between 0.6 and 0.8 was reached. The culture was then divided into two, centrifuged (7000 rpm, 15 min, 20°C) and re-suspended in 5 ml and 15 ml of liquid co-cultivation medium (CCM) containing 0.32 g l^−1^ B5 salts with B5 vitamins, 4.26 g l^−1^ MES, 1.67 mg l^−1^ 6-BA, 3.3 mM L-cysteine, 1.0 mM sodium thiosulfate, 1.0 mM DDT, and 3% sucrose. A longitudinal cut along the hilum of the seeds was then made to separate the cotyledons and the embryonic axis was excised to obtain half-seed explants. The edge of a No. 11 scalpel blade (Feather, Osaka, Japan) was dipped in 5 ml of concentrated *Agrobacterium* suspension and used to wound the explants seven or eight times at the embryonic axis. These inoculated explants were then sonicated for 20 sec and placed under a vacuum for 30 sec. Following inoculation, the cotyledons were placed in CCM solidified with 4.8 g l^−1^ agar. Co-cultivation was continued for five days at 24°C after which the hypocotyls of the explants were briefly washed in liquid shoot induction medium (SIM) containing 3.2 g l^−1^ B5 salt with B5 vitamins, 0.6 g l^−1^ MES, 1.67 mg l^−1^ 6-BA, 250 mg l^−1^ cefotaxime, 50 mg l^−1^ vancomycin, 100 mg l^−1^ ticarcillin, and 3% sucrose, pH 5.6. The explants were subsequently cultured (flat side up) on solid SIM and after 14 days were transferred to fresh SIM containing 10 mg l^−1^ DL- phosphinothricin (PPT).

After two weeks of culture on SIM with PPT, the explants were transferred to shoot elongation medium (SEM) containing 4.4 g l^−1^ MS salt with B5 vitamins, 0.6 mg l^−1^ MES, 50 mg l^−1^ asparagine, 100 mg l^−1^ pyroglutamic acid, 0.1 mg l^−1^ IAA, 0.5 mg l^−1^ GA3, 1 mg l^−1^ zeatin riboside, 250 mg l^−1^ cefotaxime, 50 mg l^−1^ vancomycin, 100 mg l^−1^ ticarcillin, 5 mg l^−1^ DL- phosphinothricin, and 3% sucrose, pH 5.7. Selection during the shoot elongation stage was carried with 5 mg l^−1^ DL- phosphinothricin. Starting at two weeks after transfer to SEM, elongated shoots were obtained. Each shoot was dipped in sterile 1 mg l^−1^ IBA for 3 min then transferred to rooting medium containing 4.4 g l^−1^ MS salt with B5 vitamins, 0.6 mg l^−1^ MES, 25 mg l^−1^ asparagine, 25 mg l^−1^ pyroglutamic acid, 50 mg l^−1^ cefotaxime, 50 mg l^−1^ vancomycin, 50 mg l^−1^ ticarcillin, and 3% sucrose, pH 5.6. Rooted plantlets were transplanted in small pots containing the soil. The plants (T_0_) were grown in a magenta jar at 24°C and an 18 h photoperiod for 1–2 weeks with an acclimation procedure and then transferred to a large pot in a greenhouse.

### Genomic DNA Analysis

Genomic DNA was extracted from the leaf tissues of soybean plants using cetyltrimethylammonium bromide. PCR was performed with KOD FX polymerase (Toyobo, Osaka, Japan) and primer sets designed to amplify regions of the *PAC* gene (5′-AATCCCTTCGCCACCTCATCCATT-3′/5′-CATGTAATGCTGCTCAAGGTACGC-3′), and *Bar* gene (5′-CATGTAATGCTGCTCAAGGTACGC-3′/5′-CATGTAATGCTGCTCAAGGTACGC-3′ yielding DNA fragments of 642 bp and 550 bp, respectively. To verify the *β-PAC* and *35S-PAC* transgenic plants, two primer sets were used to amplify the regions between the β-conglycinin promoter/35S promoter and *PAC* gene: *β-PAC,* 5′-TTGGTAACAGTAGTCCGTAC-3′/5′-TGATTCTCTCATTGCACGCC-3′ and *35S-PAC,* 5′-TCAACATGGTGGAGCACGAC-3′/5′-TGATTCTCTCATTGCACGCC-3′ yielding 736 bp and 628 bp products, respectively. To evaluate T-DNA insertions into the plant genome, additional primers were prepared to amplify the both end regions of the vector constructs used: left border (LB) to the *Bar* gene, 5′-TGGCTGGTGGCAGGATATATTGTG-3′/5′-AGACAAGCACGGTCAACTTCCGTA-3′; *PAC* gene to right border (RB), 5′-AATCCCTTCGCCACCTCATCCATT-3′/5′-TTAAACTGAAGGCGGGAAACGACA-3′. PCR amplifications were performed with KOD FX polymerase and generated 862 bp and 1310 bp amplified products, respectively. For Southern blot analyses, 30 µg of genomic DNA was digested with *Hind*III and hybridized to a *PAC* probe.

Quantitative real-time PCR (qRT-PCR) analysis to count the insertion events of transgene was performed with the CFX-96TM Real-Time system (Bio-Rad, Hercules, CA, USA). Each reaction contained 4 µl of 3.3 ng/µl DNA, 0.5 µl (10 pm/µl) of each primer, and 10 µl of iQTM SYBR® Green Supermix (Bio-Rad, Ca, USA) in a total volume of 20 µl. The amplification condition was 95°C for 3 min, 40 cycles of 10 sec at 95°C and 30 sec at 60°C, and 95°C for 10 sec, followed by the generation of dissociation curve by increasing temperature from 65 to 95°C to check for amplification specificity. The soybean genomic DNA extracted from the transgenic plant that its single *Bar* gene introgression was already confirmed was used as control (data not shown). The primers used were as follows: *Bar* F/*Bar* R, 5′-AACTTCCGTACCGAGCCGCA-3′/5′-TCGTAGGCGTTGCGTGCCTT-3′; *TUB* F/*TUB* R, 5′-TGAGCAGTTCACGGCCATGCT -3′/5′-TCATCCTCGGCAGTGGCATCCT -3′. The constitutive *TUB* was used as an internal control to normalize the amount of leaf DNAs of soybean plants.

### RNA Analysis

Total RNAs were isolated from both non-transgenic and transgenic soybean leaves and mature seeds using TRI Reagent (Sigma, St. Louis, MO) in accordance with the manufacturer’s instructions. First-strand cDNA was synthesized using Superscript™ II Reverse Transcriptase and oligo-dT (Invitrogen, Carlsbad, CA). The quantity and quality of the synthesized cDNAs were determined by spectrophotometry. To reduce systematic errors in the PCR caused by variations in cDNA quantity, all cDNA samples were diluted to 3 ng/µl and stored at −20°C until use. First-stand cDNAs were used for reverse transcriptase-PCR (RT-PCR) performed using KOD FX polymerase (Toyobo). The primers for the transgene recognized the *CrtI* gene region of recombinant *PAC* (5′-AATCCCTTCGCCACCTCATCCATT-3′/5′-CATGTAATGCTGCTCAAGGTACGC-3′).

Another qRT-PCR was performed in 96-well plates with the same CFX-96™ Real-Time system (Bio-Rad). Each reaction contained 3 µl (3 ng/µl) of cDNA, 0.2 µl (10 pm/µl) of each primer, and 10 µl SYBR® Premix Ex Taq™ (Takara) in a total reaction volume of 20 µl. The PCR conditions were 95°C for 3 min, 40 cycles of 10 sec at 95°C, 10 s at 55°C and 20 sec at 72°C, followed by the generation of a dissociation curve by increasing temperature from 65 to 95°C to check for amplification specificity. The efficiency and standard deviation of each primer were determined using Bio-Rad CFX manager v1. 6. 541.1028 on a standard curve generated from a two-fold dilution series of one sample at five dilution points for two technical replicates. Baseline and threshold cycle (Ct value) were automatically calculated using default parameters. The primers used were as follows: *Psy* F/*Psy* R, 5′-AGGATACTGGACGAGATCGA-3′/5′-AGCTTGAGGAGGTCGAAGTT-3′; *CrtI* F/*CrtI* R, 5′-CTTGAGCAGCATTACATGCC-3′/5′-CCGACCAGGTAGAGATTAGT-3′; *ACT11* F/*ACT11* R, 5′-ATCTTGACTGAGCGTGGTTATTCC-3′/5′-GCTGGTCCTGGCTGTCTCC-3′. The constitutive *Actin11* gene was used as an internal control to normalize the seed and leaf RNA levels of soybean plants [31].

### Carotenoid Extraction and HPLC Analysis

For carotenoid analysis, soybean leaves were immediately freeze-dried and disrupted in liquid nitrogen. Soybean seeds were ground to obtain a fine powder using a mortar and pestle. The powders were kept at −80°C until use. The extraction and measurement of carotenoids by high-performance liquid chromatography (HPLC) were performed as described previously by our group [32]. Briefly, carotenoids were released from the soybean samples (0.1 g) by adding 3 mL of ethanol containing 0.1% ascorbic acid (w/v), vortexing for 20 sec and placement in a water bath at 85°C for 5 min. The carotenoid extract was then saponified with potassium hydroxide (120 µL, 80% w/v) in the 85°C water bath for 10 min. After saponification, the samples were placed immediately on ice, and cold deionized water (1.5 mL) was added. Carotenoids were extracted twice with hexane (1.5 mL) by centrifugation at 1,200×*g* to separate the layers. Aliquots of the extracts were then dried under a stream of nitrogen and re-dissolved in 50∶50 (v/v) dichloromethane/methanol before analysis by HPLC.

The carotenoids were separated on a C30 YMC column (250×4.6 mm, 3 µm; Waters Corporation, Milford, MA) with an Agilent 1100 HPLC (Massy, France) equipped with a photodiode array (PDA) detector. Chromatograms were generated at 450 nm. Solvent A consisted of methanol/water (92∶8 v/v) with 10 mM ammonium acetate. Solvent B consisted of 100% methyl tert-butyl ether. Gradient elution was performed at 1 mL/min under the following conditions: 0 min, 90% A/10% B; 20 min, 83% A/17% B; 29 min, 75% A/25% B; 35 min, 30% A/70% B; 40 min, 30% A/70% B; 42 min, 25% A/75% B; 45 min, 90% A/10% B; and 55 min, 90% A/10% B. Violaxanthin, lutein, α-carotene, β-cryptoxantin and β-carotene were purchased from CaroteNature (Lupsingen, Switzerland). The carotenoid contents were calculated using HPLC peak areas of external standard calibration curves.

### Tocopherol and Phytosterol Extraction and GC-TOFMS Analysis

Determination of the tocopherol and phytosterol contents by GC-TOFMS was performed as described previously [33]. Briefly, tocopherol and phytosterol were released from the powdered soybean samples (0.05 g) by the addition of 3 mL of ethanol containing 0.1% ascorbic acid (w/v), and 0.05 mL of 5α-cholestane (10 *µ*g/mL) as the internal standard (IS), vortexing for 20 s, and incubation in a water bath at 85°C for 5 min. After removal of the samples from the water bath, 120 *µ*L of potassium hydroxide (80%, w/v) was added. After saponification, deionized water (1.5 mL) and hexane (1.5 mL) were added to each sample followed by vortexing for 20 s and centrifugation (1200×*g*, 5 min). The upper layer was then dried in a centrifugal concentrator (CVE-2000; Eyela, Tokyo, Japan). For derivatization, 30 *µ*L of *N*-methyl-*N*-trimethylsilyltrifluoroacetamide (MSTFA) with 30 *µ*L of pyridine was added and incubated at 60°C for 30 min at a mixing frequency of 1200 rpm using a thermomixer comfort (model 5355; Eppendorf AG, Hamburg, Germany).

GC-TOFMS was performed using a gas chromatograph (7890A; Agilent, Atlanta, GA) coupled to a Pegasus HT TOF mass spectrometer (LECO, St. Joseph, MI). A derivatized sample (1 *µ*L) was separated on a 30 m×0.25 mm I.D. fused-silica capillary column coated with 0.25-*µ*m CP-SIL 8 CB low bleed (Varian Inc., Palo Alto, CA). The injector temperature was 290°C, the split ratio was set at 1∶10, and the helium gas flow rate through the column was 1.0 mL/min. The temperature program was set at 200°C, followed by a 10°C/min oven temperature ramp to 310°C and a 10 min heating at 310°C. The transfer line and the ion-source temperatures were 280°C and 230°C, respectively. The scanned mass range was 50–600 *m/z*, and the detector voltage was set at 1700 V. Quantitative calculations were based on the corrected peak area ratios relative to the peak area of the IS.

### Determination of the Antioxidant Activity

Antioxidant activity was evaluated by measuring the scavenging activity of the saponified extracts on DPPH [20]. Briefly, ground soybean seed (10 mg) was extracted with 2.0 mL of methanol and different dilutions of the extracts were prepared. An aliquot of 1.0 mL of a diluted extract was vigorously mixed with 1.0 mL of freshly prepared 0.004% DPPH in methanol and held in the dark for 30 min at room temperature. The absorbance was then read at 517 nm against blanks. DPPH free radical-scavenging ability was calculated by using the following formula:

Scavenging ability (%) = [absorbance (517 nm of control) – absorbance (517 nm of sample)]/absorbance (517 nm of control)×100.

The scavenging activity of soybean seed extracts was expressed as 50% effective concentration, EC_50_ (mg/10 mL), and was obtained by interpolation from linear regression analysis. Alpha-tocopherol was used for comparison of antioxidant activity. Standard α-tocopherol and DPPH were purchased from Sigma-Aldrich Chemical Co. (St. Louis, MO, USA).

## Supporting Information

Figure S1 **Verification of** ***β-PAC*** **transformants by PCR amplification of genomic DNA extracted from T_0_ transgenic leaf tissues.** (a) *PAC* gene; (b) *Bar* gene; (c) DNA region between the β-conglycinin promoter and *PAC* gene; (d) DNA region between the left border and *Bar* gene; (e) DNA regions between the *PAC* gene and right border; PC, binary vector pβ-PAC used as a positive control; NT, non-transgenic negative control; EV, empty vector-transgenic plant; 1∼24, *β-PAC* transgenic lines.
(TIF)

Figure S2 **Verification of** ***35S-PAC*** **transformants by PCR amplification of genomic DNA extracted from T_0_ transgenic leaf tissues.** (a) *PAC* gene; (b) *Bar* gene; (c) DNA region between the 35S promoter and *PAC* gene; (d) DNA region between the left border and *Bar* gene; (e) DNA region between the *PAC* gene and right border. PC, binary vector p35S-PAC used as a positive control; NT, non-transgenic negative control; EV, empty vector-transgenic plant; 1∼19, *35S-PAC* transgenic lines.
(TIF)

Figure S3 **Tocopherol and phytosterol composition in the seeds of** ***β-PAC*** **and** ***35S-PAC*** **transgenic soybean plants.** (a) Tocopherols. (b) phytosterols. The total amounts of tocopherol and phytosterol and the levels of representative components are shown for four *β-PAC* lines and two *35S-PAC* lines. Values (µg/g dry weight) are the mean of three replicates. Error bars represent the standard deviation.
(TIF)

Table S1 **Carotenoid content and composition of** ***β-PAC*** **and** ***35S-PAC*** **transgenic soybean plants.**
(DOCX)
