# Supplementary figures and images for: Genetic Modification of the Soybean to Enhance the β-Carotene Content through Seed-Specific Expression
Source: PLoS One. 2012 Oct 31;7(10):e48287. doi: 10.1371/journal.pone.0048287 (PMC3485231; doi:10.1371/journal.pone.0048287)

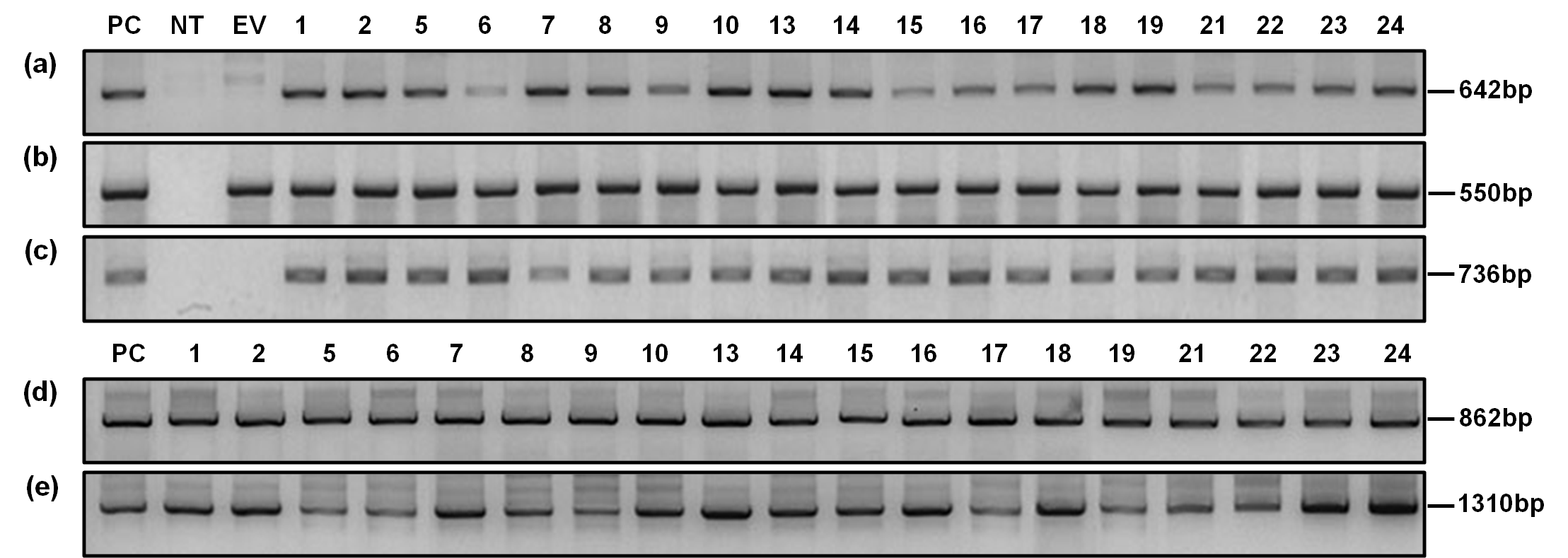

Supplement: Figure S1 — Verification of β-PAC transformants by PCR amplification of genomic DNA extracted from T0 transgenic leaf tissues. (a) PAC gene; (b) Bar gene; (c) DNA region between the β-conglycinin promoter and PAC gene; (d) DNA region between the left border and Bar gene; (e) DNA regions between the PAC gene and right border; PC, binary vector pβ-PAC used as a positive control; NT, non-transgenic negative control; EV, empty vector-transgenic plant; 1∼24, β-PAC transgenic lines. (TIF) [file pone.0048287.s001.tif]

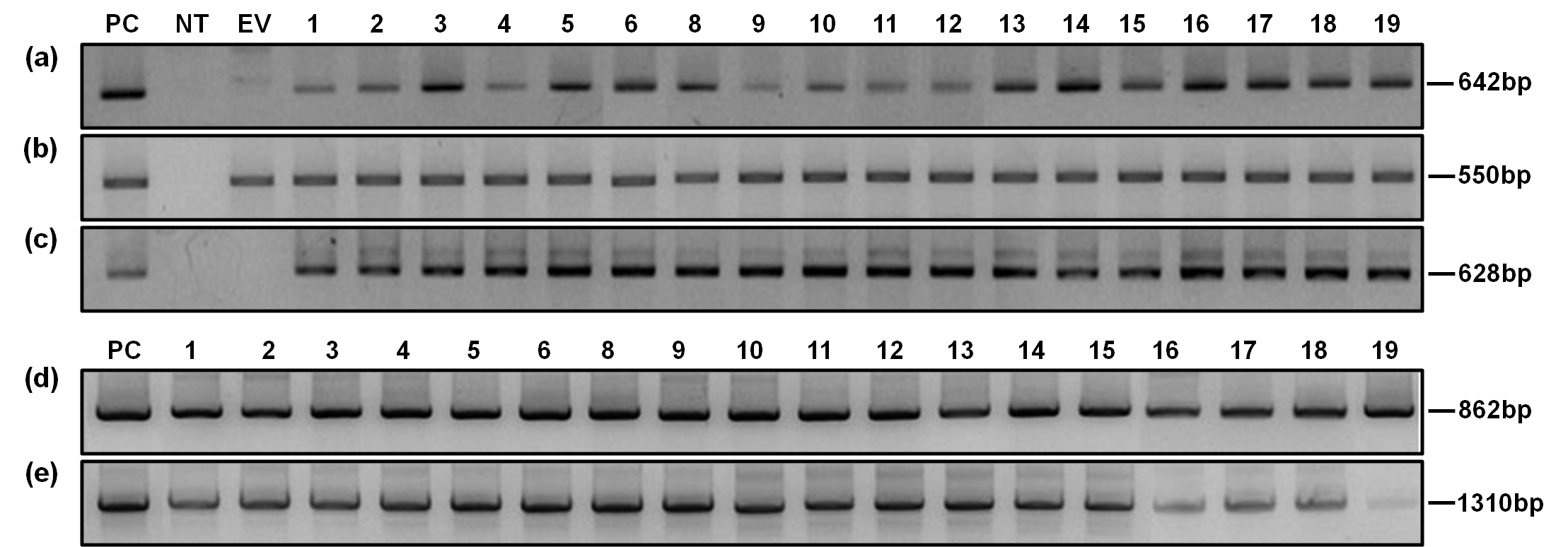

Supplement: Figure S2 — Verification of 35S-PAC transformants by PCR amplification of genomic DNA extracted from T0 transgenic leaf tissues. (a) PAC gene; (b) Bar gene; (c) DNA region between the 35S promoter and PAC gene; (d) DNA region between the left border and Bar gene; (e) DNA region between the PAC gene and right border. PC, binary vector p35S-PAC used as a positive control; NT, non-transgenic negative control; EV, empty vector-transgenic plant; 1∼19, 35S-PAC transgenic lines. (TIF) [file pone.0048287.s002.tif]

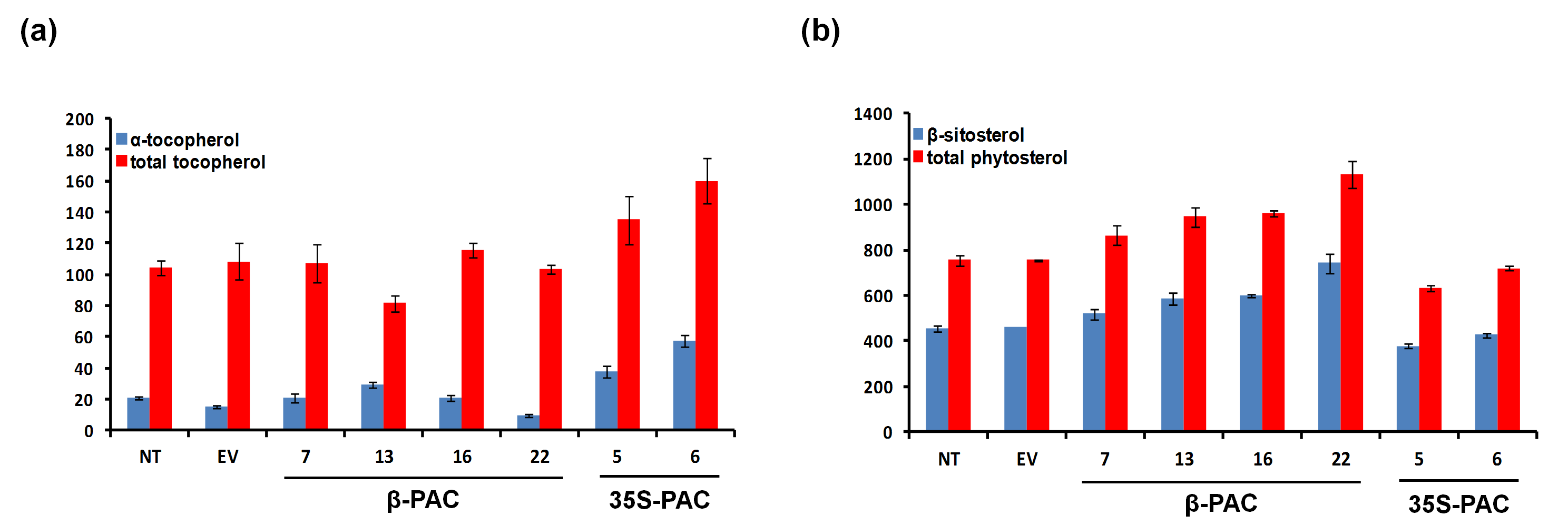

Supplement: Figure S3 — Tocopherol and phytosterol composition in the seeds of β-PAC and 35S-PAC transgenic soybean plants. (a) Tocopherols. (b) phytosterols. The total amounts of tocopherol and phytosterol and the levels of representative components are shown for four β-PAC lines and two 35S-PAC lines. Values (µg/g dry weight) are the mean of three replicates. Error bars represent the standard deviation. (TIF) [file pone.0048287.s003.tif]
