# Supplementary material for: Genetic Modification of the Soybean to Enhance the β-Carotene Content through Seed-Specific Expression
Source: PLoS One. 2012 Oct 31;7(10):e48287. doi: 10.1371/journal.pone.0048287 (PMC3485231; doi:10.1371/journal.pone.0048287)
Supplement: Table S1 — Carotenoid content and composition of β-PAC and 35S-PAC transgenic soybean plants. (DOCX) [file pone.0048287.s004.docx]

**Table S1. Carotenoid content and composition of *β-PAC* and *35S-PAC* transgenic soybean plants.**

Data are expressed as mean (μg/g dry weight) ± SD from three independent experiments using mature seeds and leaves of individual T_2_ plants.
